# Challenges faced and countermeasures implemented by county-level hospitals in China

**DOI:** 10.7189/jogh.16.03011

**Published:** 2026-05-22

**Authors:** Juanjuan Yang, Wei Jiang

**Affiliations:** 1 Department of Health Management, The Second Affiliated Hospital of Xi’an Jiaotong University, Xi’an, China; 2 The Comprehensive Breast Care Center, The Second Affiliated Hospital of Xi’an Jiaotong University, Xi’an, China

## Abstract

China’s rapid economic growth and urbanisation have led to a sustained decline in the population of its rural and county areas, posing a significant challenge to its healthcare system. This has especially impacted county-level hospitals in the country’s western regions, which have been facing issues such as declines in quality of medical care and shortages of medical professionals and medical equipment. Areas such as cardiovascular diseases, chronic diseases, mental health, oncology, and paediatrics are especially underserved in these areas. To address these challenges, it is essential for China to adopt strategies targeted at improving diagnostic and treatment capabilities, attracting medical talent, integrating medical resources, and increasing government investment.

China’s economic development and urbanisation have invertedly led to a continuous decline in the population of rural and county areas, indirectly placing a considerable burden on the country’s healthcare system. According to the 2020 census data, 546 out of 1874 counties in China have a permanent resident population of less than 200 000, with 389 being located in its western region [1]. This trend has intensified in recent years. Simultaneously, shifts in local economic conditions and population density, and the closure of a number of hospitals due to population drops have impacted the efficiency of county-level hospitals [2], with only 54.3% of those in the western regions of China meeting recommended service capacity standards [3].

## THE COUNTY-LEVEL HOSPITALS IN WESTERN CHINA ARE FACING MULTIPLE CHALLENGES

The aforementioned population decline, combined with the expansion of high-speed railways, has enabled rural residents to seek better medical care in larger cities, thereby reducing the number of patients visiting local hospitals. This shift impacted surgical staff’s skills, proficiency, and decision-making capacities, as they can only be improved and maintained through continuous hands-on practice [4-6], and consequently led to declines in the efficiency and quality of medical care, particularly in the surgical departments [7]. According to a 2024 report, 22.3% in county-level hospitals in China of discharged patients underwent surgery, representing a downward trend from previous periods [3].

A second challenge came in the form of a critical shortage of medical professionals, whereby a significant interaction effect was observed between the number of hospital healthcare technicians and number of healthcare technicians per 1000 people on county-hospitals efficiency growth in China [8]. Specifically, there was an average of 162 medical doctors per county hospital in the western region of China in 2024, which is far below the national average of 220 [3]. Urbanisation and suboptimal personnel management have driven young medical graduates to move to large cities, where the expansion of tertiary hospitals provides better employment opportunities [9,10]. Simultaneously, western regions often lack specialised medical departments: for instance, more than 30% of county-level hospitals in some provinces lack psychiatry departments [3]. Similarly, departments of neurosurgery, cardiothoracic surgery, and urology, among others, are unavailable in certain county hospitals [3]. There is also a noticeable lack of essential medical equipment in these contexts, with some hospitals lacking mammography units, despite the Chinese government implementing breast cancer screening programmes for women [11], which limits the capacity for early diagnosis and treatment.

## THE INSUFFICIENT SERVICE CAPACITY OF COUNTY-LEVEL HOSPITALS EXACERBATED INEQUALITIES IN ACCESS TO BASIC HEALTHCARE

The insufficient service capacity of county-level hospitals has created structural disadvantages for rural residents, limiting their access to high-quality medical services and exacerbating inequality. Due to population ageing and the migration of young people to urban areas, China’s counties mainly remain home only to the elderly and primary/secondary school students – two groups facing distinct sets of healthcare conditions. A study of 63 205 secondary school students in western China identified that the prevalence rates of depression and anxiety disorders were 23.0% and 13.9%, respectively [12]. Conversely, common diseases among the elderly include cardiovascular and cerebrovascular diseases, metabolic and endocrine disorders, bone and joint diseases, chronic obstructive pulmonary disease, and malignant tumours [13,14]. In 2022, the estimated number of new malignant tumour cases and the estimated number of deaths from all tumours in rural areas of China were 1.92 million and 1.17 million, respectively, with higher mortality rate in rural than in urban areas [15]. However, only 61.49% and 79.16% of county-level hospitals, respectively, have oncology and psychiatry departments, while only 46.96% and 65.66% of psychiatry and pathology departments meet these basic standards. Furthermore, over 35% of county-level hospitals have not yet implemented immunohistochemistry, molecular biology-assisted diagnosis, or special staining pathological techniques for tumours [3]. Consequently, patients with mental disorders or tumours often have to travel long distances to receive professional treatment, which often comes with significant financial expenses. This can also lead to delayed treatment for conditions such as stroke or trauma, resulting in hikes in preventable morbidity and mortality, as well as catastrophic health expenditures.

## THE CHINESE FOVERNMENT AND HEALTHCARE POLICYMAKERS MUST ADOPT A SERIES OF STRATEGIES IN RESPONSE TO THESE CHALLENGES

### Short-term actions (0–2 years)

A first step in addressing these challenges can be the improvement of diagnostic and therapeutic capabilities for prevalent and critical diseases in rural regions, with priority areas including cardiovascular diseases, trauma, chronic diseases, mental health, oncology, and paediatrics. To achieve this, the government has transferred doctors from tertiary to county-level hospitals to assist in establishing specialised departments and improving local staff’s clinical skills [16,17]. County-level hospitals should also strengthen their training of doctors through standardised residency programmes and clinical rotations in tertiary hospitals. Given the differences in departmental structures between county-level hospitals and tertiary hospitals, the latter should develop individualised training plans for doctors in the former. Telemedicine platforms can further allow medical professionals to provide real-time guidance to county-level hospitals, thereby facilitating fairer access to healthcare services. To motivate medical professionals, county-level hospitals and their collaborating tertiary institutions should implement performance-based reform measures and increase rewards beyond a fixed salary. Assessment metrics should include the coverage rate of the specialty, the threshold of surgical volume, stroke door-to-needle times, and the completion rate of referrals.

Second, China should strive to strengthen its workforce by expanding the free targeted medical student training programmes which recruit rural students from counties and provide them with free medical education, while mandating them to work in county-level hospitals after graduation in return. Accessible resources and higher salaries can further improve medical staff’s job satisfaction and reduce burnout [18]. Besides this, providing high-quality educational resources for their children in less developed regions may also attract young professionals. The Chinese government has already stipulated that scientific research papers should not be a mandatory requirement for applying for professional titles for healthcare professionals [19]. This requirement should be eliminated for medical doctors in county-level hospitals, with promotions solely based on clinical competences. These measures fall under the purview of county-level hospitals and health bureaus. Here, assessment metrics should include the retention rate and the turnover rate of medical doctors.

### Medium-term actions (3–5 years)

It is also necessary for China to integrate existing county-level hospital resources in western regions. Most counties in the country have at least three public hospitals: a people's hospital, a maternal and child health hospital, and a traditional Chinese medicine hospital. While the latter two specifically focus on areas such as obstetrics, gynaecology, paediatrics, and traditional Chinese medicine, they often overlap with the former in providing these services. Integrating these three institutions into a single comprehensive hospital could help optimise the utilisation of resources. For example, maternal and child health hospitals can be merged into the obstetrics, gynaecology, paediatrics, and preventive healthcare departments of people’s hospitals, while the traditional Chinese medicine hospital can be integrated into the traditional Chinese medicine department and department of integrated traditional Chinese and western medicine of people’s hospitals. This would facilitate equipment sharing, eliminate redundancy, and alleviate physician shortages. The county health bureau should lead this effort by first launching a one-year a feasibility study; if successful, a subsequent merger could be advanced and finalised within three to five years.

Increasing government investment should also be a priority: national ministries should allocate special funds to upgrade outdated equipment and purchase currently scarce, but essential devices, such as digital subtraction angiography machines and mammography units. Financial subsidies should also be provided for socially necessary, low-fee diagnostic tests. The main financial source here are the government’s earmarked readiness grants, while the related actions are the responsibility of national ministries. The assessment metric could be the completion of the procurement of critical equipment within two years and replacement of all obsolete equipment within three to five years.

## EXPERIENCES FOR OTHER LOW- AND MIDDLE-INCOME COUNTRIES

Other low- and middle-income countries face similar structural challenges, including rural depopulation, poor or unequal distribution of clinical staff, the over-concentration of specialised services in urban centres, and underutilisation of district-level facilities. For instance, 80% of medical specialists in India reside in urban settings, while nearly 70% of the population lives in rural areas, where only 13% of the people have access to primary health centres [20,21]. Most district hospitals in the Indian public sector operate below expectations in terms of availability, accessibility, and quality, with variations across districts in terms of the number of beds, the availability of equipment, the provision of services, and population coverage [22]. In Brazil, healthcare infrastructure in the Amazon and the northeast region remains underdeveloped compared to the rest of the country, which particularly affects indigenous populations [23].

China’s experiences include formulating national policies to ensure equitable access to basic medical services, promoting the development of specialised department in county-level hospitals, setting up medical scholarships for studying in rural regions, establishing telementoring networks, and implementing performance reforms. These experiences could provide valuable references for other low-and middle-income countries. However, the allocation of medical education resources, integration strategies, and government investment must be tailored to the specific circumstances of each setting.

## CONCLUSIONS

The sustained decline in the population of rural and county areas has posed significant challenges to China’s county-level healthcare system. These challenges could be overcome through targeted policy adjustments, talent cultivation, resource integration, and increased investment, with the resulting insights providing valuable data for other low-and middle-income countries.
